# Solar-Driven Soil Remediation along with the Generation of Water Vapor and Electricity

**DOI:** 10.3390/nano12111800

**Published:** 2022-05-25

**Authors:** Xiaoting Liu, Zhe Wang, Hanxue Liang, Yuanyuan Li, Tianfu Liu, Qiang Guo, Liru Wang, Ya’nan Yang, Nan Chen

**Affiliations:** 1 Key Laboratory of Cluster Science, Ministry of Education of China, School of Chemistry and Chemical Engineering, Beijing Institute of Technology, Beijing 100081, China; xiaoting_liu2@126.com (X.L.); wzhe1214@gmail.com (Z.W.); yyli3478430@163.com (Y.L.); 3120185630@bit.edu.cn (Q.G.); example_wlr@163.com (L.W.); yangyanan310@163.com (Y.Y.); 2 Key Laboratory of Photoelectronic/Electrophotonic Conversion Materials, School of Chemistry and Chemical Engineering, Beijing Institute of Technology, Beijing 100081, China; 3 Yangtze Delta Region Academy of Beijing Institute of Technology, Jiaxing 314019, China; 4 Tianjin Key Laboratory of Biosensing and Molecular Recognition, Research Centre for Analytical Sciences, College of Chemistry, Nankai University, 94 Weijin Road, Tianjin 300071, China; lhxsdb@163.com

**Keywords:** solar-driven, soil, interfacial water evaporation, streaming potential, soil remediation

## Abstract

As a renewable energy source, solar energy has become an important part of human energy use. However, facilities utilizing solar energy are often complex and technically difficult, and preparation equipment and materials are expensive, while these equipment and materials often cause new environmental pollution. Soil, which exists in large quantities on the earth’s surface, is an inexhaustible natural material with loose and stable properties. Due to the specificity of its composition and microscopic form, the soil has an inherent advantage as a medium for solar thermal and photovoltaic conversion. Here, we built an integrated solar energy utilization system, the Integrated Soil Utilization Module (ISUM), integrating multi-functions into one hybrid system, which enables solar-driven water vapor and electricity generation and soil remediation. The evaporation rate of the soil represented by the rocky land was 1.2 kg·m^−2^·h^−1^ under 1-sun irradiation with evaporation induced voltage of 0.3 V. With only seven days of continuous exposure to sunlight, the removal of heavy metal ions from the soil reached 90%, while the pH was raised to near neutral. The combined application of readily available natural soil with solar energy not only demonstrates the potential of a soil for solar desalination and power generation, but in addition, solar-driven interfacial evaporation provides an energy-efficient, environmentally friendly, and sustainable method for purifying heavy metal and acid-contaminated soil.

## 1. Introduction

The world energy consumption structure is unreasonable, and the energy security system is fragile. It has become a global consensus to take various measures such as energy diversification and open source and cost reduction to achieve sustainable development of energy. Mankind has been deeply aware of the limitations and inefficiency of high-grade energy, in reducing the use of high-grade energy at the same time, it is particularly important to comprehensively improve the utilization of renewable low-grade energy. Solar energy is a clean, abundant renewable low-grade energy on Earth [1,2,3,4]. The use of solar energy has two ways of photothermal conversion and photoelectric conversion. Among them, photothermal conversion is mainly manifested as solar-driven interfacial evaporation, which has so far become one of the most promising solar thermal technologies due to its potential applications in purification, distillation, desalination, and power generation [5,6,7]. Considerable work has been done in developing reliable and efficient light-absorbing materials for photothermal conversion, including various carbonaceous materials (graphene [8,9,10,11,12,13,14], carbon nanotube [15], carbon nanoparticles [16,17], biochar [18,19]), plasmonic-based metals [20,21,22,23], inorganic semiconductors [24,25,26], organic polymers [27]. In addition, photovoltaic conversion, specifically photothermal conversion, is also manifested in the generation of electricity through water evaporation. Solar-driven interfacial evaporation can induce water molecules to generate electricity while desalinating. Thus, hybrid systems combining solar desalination and electricity generation have shown the potential of solar water evaporation technology for freshwater and green energy production in recent years [28,29,30,31,32]. However, as most photothermal materials are non-renewable products currently, the development of natural alternatives has gained increasing momentum in recent years [33]. In the Solar-driven interfacial evaporation, we found that the morphology of water in photothermal materials is very similar to that of water in the soil, which can be classified into four types: hygroscopic/film water, capillary water, and gravity water [9], and combined with the widespread presence of soil evaporation in nature, these phenomena reveal that soil is also an optional medium capable of absorbing solar thermal energy and achieving photothermal conversion [34].

It is also of concern that frequent global human activities have made the problem of soil contamination, on which people depend, more and more serious. For example, heavy metal contamination or soil acidification not only affects can be toxic to the normal growth and development of crops, but also affects the safety of consumption of agricultural products and the physical health of humans/organisms due to residues, which results in significant environmental degradation over time [35], and eventually cause a global ecosystem balance crisis. Currently, common soil remediation techniques include physical techniques, chemical methods, and bioremediation [36]. However, these techniques are not conducive to larger-scale replication because they are time-consuming, environmentally unfriendly, costly, and resource-consuming. Previously, petroleum compounds have been removed from contaminated soil by thermal desorption of low temperature, which uses heat to evaporate volatile pollutants from the soil, but it is limited to removing only volatile pollutants from the soil. Thus, we think of using solar-driven interfacial evaporation to produce water vapor and remove soluble contaminants from the soil [37]. As with the drenching method, water in the soil can release soluble heavy metal cations and acid ions from the functional groups on the surface of soil particles and enrich them on the soil surface with the water transport during the solar-driven transport from bottom to top, thus achieving the remediation of contaminated soil. The remediation method is based on the concept of solar-driven interfacial evaporation, allowing a soil washing process without additional energy consumption and pollution. In this regard, we propose a device called Integrated Soil Use Module (ISUM), which drives water purification and water-induced power generation through solar-driven interfacial evaporation, in parallel with soil remediation.

Herein, we present a newly designed soil-based hybrid system, the Integrated Soil Utilization Module (ISUM), which generates electricity while evaporating water driven by solar energy. This is the first use of soil as a photothermal material in the field of energy. Furthermore, solar-driven interfacial water evaporation was found to simultaneously enable remediation of contaminated soil, which can be considered a novel and environmentally sustainable soil remediation technology for the treatment of contaminants such as heavy metals, and the remediation process is environmentally sustainable. In solar-driven soil contaminant treatment, water molecules in the soil flow upward under the action of photothermal heat and simultaneously transport heavy metal cations and hydrogen/acid ions, achieving efficient enrichment of heavy metal or acid contaminants to the soil surface in the short term. This multifunctional soil-based ISUM system can not only achieve an evaporation rate of 1.2 kg·m^−2^·h^−1^ and obtain a stable induced voltage output of 0.3 V but also can effectively remediate soil pollution, which can be described as “three eagles with one arrow”. The ISUM provides a new equipment platform for the development of sustainable and environmentally friendly water treatment systems, low-grade energy harvesting, and contaminated soil decontamination.

## 2. Materials and Methods

### 2.1. Pretreatment of Soil

Equal amounts of the six soils were baked in an electric oven at 60 °C for 10 h until dry and subsequently ground with a mortar. Finally, sieve six soils into uniform particles with 10-, 30-, 60-, and 100-mesh sieve (Figure S1).

### 2.2. Preparation of Soil Contaminated by Heavy Metals or Acid

500 mL aqueous solution containing Zn^2+^ and Cd^2+^ at a concentration of 1000 mg·L^−1^ was added to 500 g of soil, and the mixture was stirred evenly before drying. The concentrations of Zn^2+^ and Cd^2+^ in soil measured by ICP were 142.5 mg·kg^−1^ and 148.6 mg·kg^−1^, respectively. Table S1 shows energy dispersive spectrum analysis of heavy metal contaminated soil. Forty milliliters of 1 mol·L^−1^ hydrochloric acid solution was added to 120 g of soil and stirred evenly to obtain an acidic soil with a pH value of 3.

### 2.3. ISUM Manufacturing

Several bottomless acrylic square frames were customized to hold the soil to maintain its shape, measuring L5 × W5 × H6 cm^3^. In addition, the electrodes used in the test were titanium mesh (100 mesh) of the same size as an acrylic square frame and connected to the meter with silver wire (0.4 mm in diameter) of 10 cm in length. Cut the right size filter paper and place it on the bottom surface of the soil to obtain a uniform infiltration rate.

### 2.4. Characterization

Soil morphology and energy-dispersive X-ray spectrometry were carried out on an SEM (Zeiss SUPRA TM 55 SAPPHIRE, Oberkochen, Germany). The X-ray diffraction was tested on a Netherlands 1710 diffractometer with a Cu Kα irradiation source (λ = 1.54 Å). The TG was measured by Rigaku TG-DTA 8122. The absorbance was obtained by a UV/VIS/NIR spectrometer (UH4150 Spectrophotometer, Beijing, China). The light intensity is measured with a Solar Power Meter (SM206-SOLAR, Shenzhen, Guangdong, China), and a High-Power Xenon Light Source (MC-X301B) is used to simulate sunlight. Optical photos were obtained by photon microscope (TD-TZG5-4KC-4K1506). Current and voltage were measured by using a Keithley 2400 multimeter. The particle size distribution was measured by using a laser particle size analyzer (Mastersizer 2000, Malvern, UK). The surface temperature was acquired by Thermal Imagers and Infrared Cameras (FLUKE-TiS75+). The zeta potentials were performed on a potentiometer (Zetasizer Nano ZS90, Malvern Instruments Limited, Malvern, UK). Ion concentrations were obtained from ICP-OES (PerkinElmer 8300, Waltham, MA, USA).

## 3. Results

### 3.1. Composition and Characteristics of Six Typical Soils

**Figure 1:**
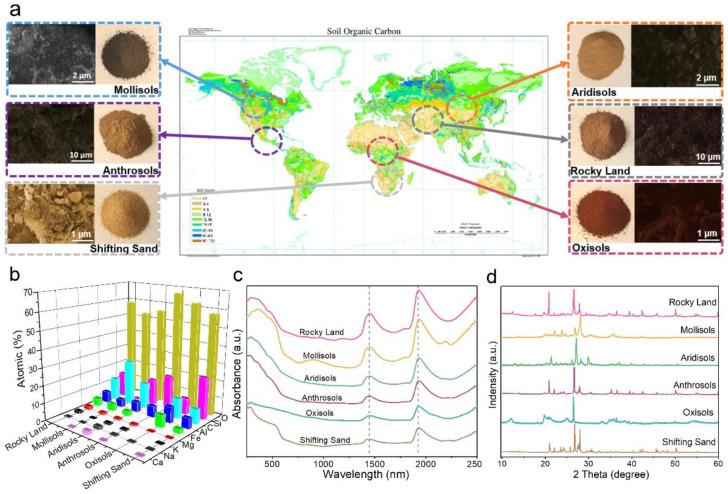
(**a**) Photos and scanning electron microscope (SEM) images of six soils and their main distribution in the global soil organic carbon map; (**b**) Energy dispersive spectrum (EDS) analysis of the six soils (the atomic percentage of the major elements); (**c**) X-ray diffraction spectra (XRD) of the six soils; (**d**) Absorbance spectra in the wavelength range of 250–2500 nm.

Six typical soils on Earth were selected as rocky land, anthrosols, aridisols, oxisols, mollisols and shifting sand (Figure 1a). According to the global soil distribution map updated on the website of USDA in 2005 [38], the specific distribution of these six soils in the global soil organic carbon content map [39] is shown in Figure 1a, indicating that the organic carbon content of these soils is low, so we mainly analyze the inorganic substances contained in them here. These soils possess similar microscopic morphology as seen from the optical photos, SEM photos and photon microscope photos of Figure 1a and Figure S1. The EDS elemental analysis in Figure 1b shows that the major elements in the six soils are O, Si, C, Al, and Fe, while the content of Mg, K, Na, and Ca is low. In addition, the general material composition of the soil (Figure 1c) and the content of bound water in the soil (Figure S2) were obtained by XRD and Thermogravimetry (TG). Since the composition of shifting sand is very stable, there is no loss of bond water mass in the low-temperature range for TG measurement. Refined characterization of the six soils (rocky land, oxisols, aridisols, the shifting sand, anthrosols, and mollisols) indicates that its chemical formula can be written as Al_2_O_3_·0.37Fe_3_O_4_·4.03SiO_2_·4.03H_2_O, Al_2_O_3_·0.52Fe_3_O_4_·2SiO_2_·4.69H_2_O, Al_2_O_3_·0.28Fe_3_O_4_·5.03SiO_2_·4.42H_2_O, Al_2_O_3_·0.28Fe_3_O_4_·8SiO_2_, Al_2_O_3_·6.05Fe_3_O_4_·5.05H_2_O and Al_2_O_3_·0.77Fe_3_O_4_·5.6SiO_2_·9.4H_2_O, respectively. The absorbance of six soils in the wavelength range of 250–2500 nm was further tested using UV-VIS-NIR spectroscopy as shown in Figure 1d, indicating that several soils have good absorption of sunlight, which also indicates that the soil has a facilitative effect on water evaporation. The primary focus here is on the effect of water transport rate, particle size, thickness, and soil water morphology on water evaporation performance.

### 3.2. Water Vapor Generation Performance of ISUM

**Figure 2:**
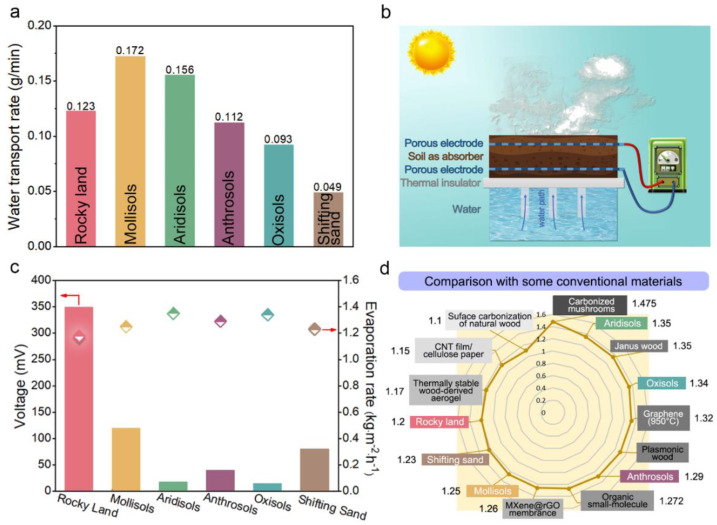
(**a**) Water transport rates in the six soils; (**b**) Structure of ISUM for solar desalination and electricity generation; (**c**) Comparison of induced voltage and water evaporation rate for the six soils (the bars represent voltage and the diamonds represent evaporation rate; the left arrow refers to bars and the right arrow refer to the diamonds); (**d**) Comparison of water evaporation rates of some typical carbon-based materials under the same sun irradiation.

Figure 2a shows the rates of water transport in several of these soils. Mollisols have the largest transport rate, shifting sand has the smallest, and there is little difference between the values for the other soils. The rocky land in ISUM is in indirect contact with water through 8 mm diameter absorbent cotton bars, and the rate of water intake is regulated by the number of bars to allow an optimal ratio of capillary water in the soil (Figure S3). A layer of filter paper was placed between the soil and the absorbent cotton bars to prevent soil particles from blocking the cotton bars and thus affecting its water absorption effect. Two inert metal mesh electrodes were buried at different heights in the soil, and the electrodes were led outward by silver wires. The solar simulator was set to 1-sun irradiation and the water evaporation rates were tested for different types of soils and for rocky land with different particle radii. The effects of factors such as sun irradiation intensity and soil thickness on the water evaporation rate were also looked at. Figure S4 shows the surface temperature of these six soils during evaporation, which is in good agreement with their water evaporation rates (Figure 2c). As can be seen in Figure 2c, the water evaporation rates for rocky land, mollisols, aridisols, anthrosols, oxisols, and shifting sand are 1.2, 1.25, 1.35, 1.29, 1.34, and 1.23 kg·m^−2^·h^−1^, respectively, are not very different from each other and are similar to typical 3D carbon-based materials in Figure 2d [18,22,40,41,42,43,44,45,46], indicating that the evaporation performance of soil as a photothermal material is fine. As shown in Figure 2c, the induced voltage signals generated by ISUMs constructed from different soils show significant differences, which are not positively correlated with evaporation rates. In addition to the effect of evaporation rate, the induced voltage is also related to various other factors. Qu’s group [47] reported that the zeta potential is related to the induced voltage. Therefore, evaporation rate and Zeta potential are all factors that affect the magnitude of the induced voltage, but they are not the decisive factors. In the test of induced voltage, although our preprocessing procedure is the same, the results are not completely consistent with the theory. This may be related to different nanochannels formed by the uniformity of particle size distribution of the six soil particles, diverse soil morphology, and hydrophilic properties [47]. Here we mainly analyze the viscosity of the soil itself. The higher the soil viscosity and the smaller and more pores can result the stronger the tendency of the horizontal flow of water inside the soil than vertical flow and the smaller the potential difference between the upper and lower electrodes in the soil, therefore the smaller the electrical signal. Among the six selected soils, oxisols exhibit the highest viscosity, followed by aridisols, anthrosols, mollisols, and rocky land, while shifting sand is basically not viscous. Considering water evaporation rate and induced voltage together, the rocky land was selected among the six soils for the follow-up study.

**Figure 3:**
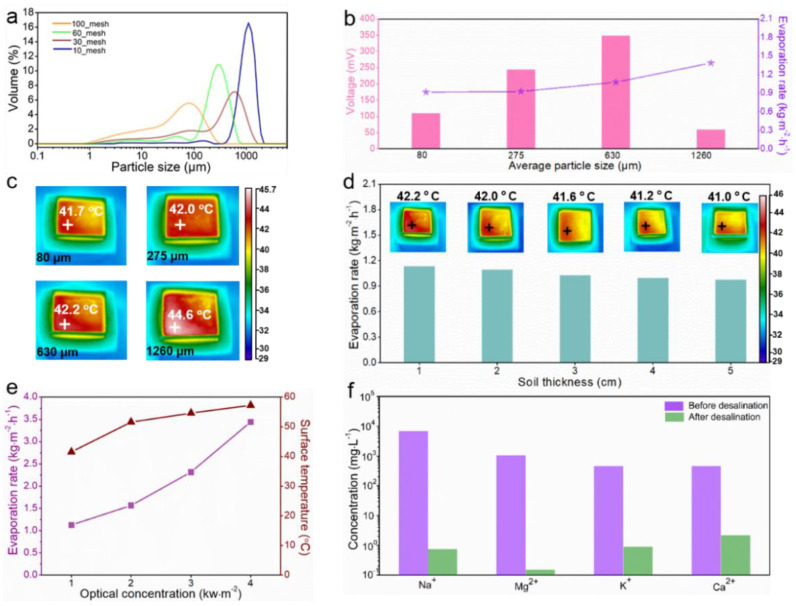
(**a**) Particle size distribution graph of 10_, 30_, 60_, and 100_mesh rocky land; (**b**) Induced voltage and water evaporation rate of rocky land with different particle sizes; (**c**) The surface temperature of different particle sizes under 1-sun irradiation for 1 h; (**d**) The water evaporation rate in rocky land with various thicknesses and the surface temperature of different thicknesses under 1-sun irradiation for 1 h; (**e**) The water evaporation rate in rocky land under 1 to 4-sun irradiation; (**f**) Concentration measurement of four main ions in seawater sample before and after desalination.

The rocky land was sieved into uniform particles of different sizes using 10-, 30-, 60-, and 100-mesh sieves, respectively, and their average particle size distributions were obtained by a laser particle size analyzer, as shown in Figure 3a. Figure 3b,c further reveals the relationship between the water evaporation rate and the average particle size of the rocky land (see Figure S6a,b in the Supporting Materials for more details). The water evaporation rate increased slightly with the increase of the average particle size, where the water evaporation rate of the rocky land with a particle size of 80 μm was only 0.92 kg·m^−2^·h^−1^, while the water evaporation rate of the rocky land with a particle size of 1260 μm was as high as 1.38 kg·m^−2^·h^−1^. This is because the larger the particle size, the larger the pores between the agglomerates of the soil particle size, and the less capillary action, so the water holding capacity is low, making the water evaporation rate faster. The induced voltage was also measured for four particle sizes of rocky lands, where the best performance was obtained for the particle size of 630 μm. It was further developed that (Figure 3d) the water evaporation rate decreases slightly as the thickness of the soil used (distance between the two inert metal grids) increases, so there is little negative correlation between soil thickness and water evaporation rate. As seen in Figure 3e, the water evaporation rate and soil surface temperature increased with increasing sun irradiation. The water evaporation rate increased to 3.5 kg·m^−2^·h^−1^ at 4-sun irradiation when the soil surface temperature also increased to about 57 °C. ICP-OES was used to detect the concentrations of four conventional ions in water to evaluate the water quality before and after desalination. After desalination, the concentration of Na^+^, Mg^2+^, K^+^, and Ca^2+^ was reduced from 6900, 1050, 461, and 460 mg·L^−1^ to 0.756, 0.153, 0.905, and 2.2 mg·L^−1^ (see Figure S7 in the Supporting Materials for more details). The concentration of salt was far below the WHO drinking water standard, as shown in Figure 3f [48]. Additional experiments have confirmed that water evaporation in ISUM can be sustained and stable for more than 10 h (see Figures S8–S10 in the Supporting Materials for more details).

### 3.3. Electricity Generation Performance of ISUM

**Figure 4:**
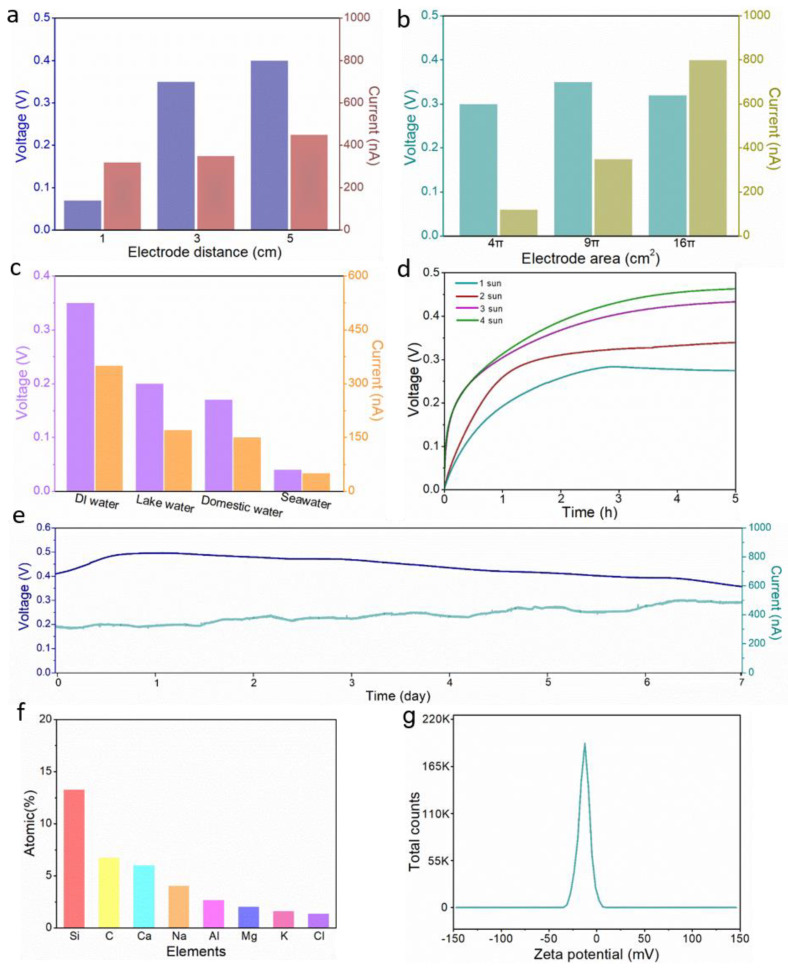
(**a**) Output voltage and current of different electrode distances; (**b**) Output voltage and current of different electrode areas; (**c**) The effect of water type on the induced electrical signals (ion concentration from low to high: deionized water < lake water < domestic water < seawater; lake water was taken from the Beijing Institute of Technology Lake, and seawater was taken from the Yellow Sea of China); (**d**) Induced voltage of ISUM under different sun irradiation; (**e**) Stability test of ISUM under 1-sun irradiation; (**f**) EDS analysis of dissolved rocky land supernatant; (**g**) Zeta potential of rocky land.

The induced electrical signals generated by ISUM in soil with two reticulated electrode distances of 1, 3, and 5 cm at 1-sun irradiation are shown in Figure 4a. The induced voltage gradually increases with increasing electrode distance and the current does not change much, which is attributed to the large difference in hydrated hydrogen proton concentration between the electrodes, resulting in a large potential difference at high capillary heights. Similarly, the induced current is proportional to the electrode area (i.e., titanium mesh area), while the voltage hardly changes, as shown in Figure 4b. The system resistance of ISUM decreases with increasing electrode area, so the cumulative effect of the inter-electrode potential difference at constant distances is essentially the same. As shown in Figure 4c, the induced voltage and induced current generated by deionized water, lake water, domestic water, and seawater in ISUM are compared respectively. The induced electrical signal in ISUM gradually decreases with increasing ion concentration in water, which is mainly attributed to the double electric layer (EDL) theory. Due to the presence of EDL at the solid-liquid interface, according to previous studies [47], flow-induced voltages and currents are generated when water flows through channels formed by surface charged soil particles driven by external evaporation and capillary action. As shown in Figure 4d, the steady-state induced voltage increases with the increase of sun irradiation intensity. The results show that with the increase of solar radiation intensity, the water evaporation rate of soil increases, resulting in a rise in the voltage obtained (Figure S12). The stability of the power generation process in an open environment is also verified, as shown in Figure 4e, where the induced voltage can remain stable for at least seven days.

The atomic percentage of soluble salt elements dissolved in the rocky land detected by EDS in Figure 4f shows that the rocky land contains a small number of soluble metal cations. According to the flow-potential principle, the induced voltage depends on the directional flow of water as well as the Zeta potential of the material surface. As shown in Figure 4g, the zeta potential of the rocky land particles is –12.8 mV, indicating that their surfaces are negatively charged under neutral conditions (see Figure S13 in the Supporting Information for details of the surface zeta potentials of other selected soils). When water evaporation-driven molecules and ions pass through capillary channels formed by negatively charged soil particles on the surface, OH^−^ is repelled in the channels, while the antagonistic ion H_3_O^+^ in the EDL can follow the fluid from the bottom to the top, accompanied by the driving force of evaporation and capillary action. Eventually, the high potential of the top electrode and the low potential of the bottom electrode produces a voltage difference (induced voltage and current values of 0.3 V and 200 nA for a single ISUM, respectively, as shown in Figure S14).

**Figure 5:**
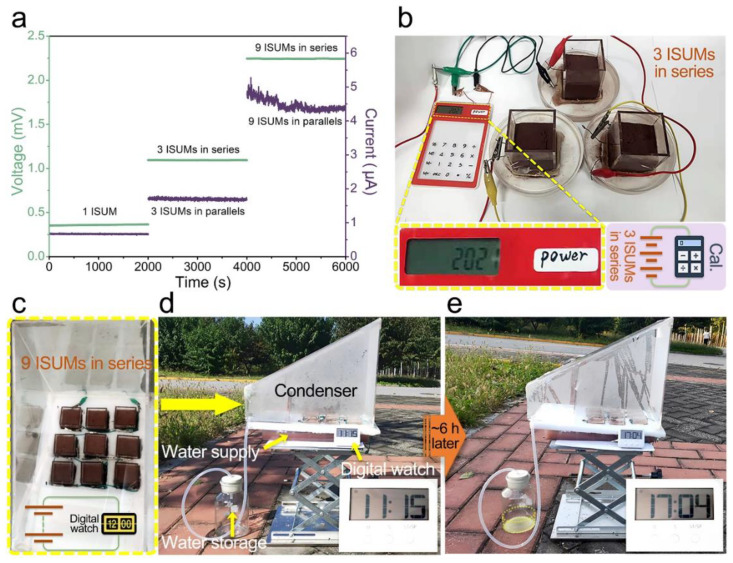
(**a**) Induced voltages and currents of 1, 3, 9 ISUMs composed of rocky land in series and parallel connection; (**b**) Integrated 3 ISUMs can power an electronic calculator; (**c**) The top view of 9 ISUMs in series; (**d**) Photo of an integrative device in sunny outdoor practical application at 11:15 (the small arrows refer to the parts of device) and after 6 h (**e**) of sunlight exposure at 17:04.

The induced voltages and currents of independent ISUM and 3 or 9 ISUM series and parallel arrays also roughly conform to Ohm’s law, with 9 ISUMs series producing an induced voltage of up to 2.3 V and 9 ISUMs parallel producing an induced current of 4.5 µA, as shown in Figure 5a. The 3 ISUMs in Figure 5b can power a small calculator in the series, indicating that the ISUMs, a low-level energy harvesting device, are sufficient to meet the power requirements of some commercial electrical products. The actual operation of the ISUM arrays in Figure 5c obtained by series connection under natural sunlight is demonstrated, in which 3 × 3 arrays of ISUMs were fabricated using rocky land, where each ISUM consists of soil with a volume of L5 × W5 × H6 cm^3^. The ISUM array is placed inside a homemade transparent acrylic box under sunlight, and the seawater to be purified is delivered to the bottom of the acrylic box, as shown in Figure 5d,e. The pure water produced by the solar drive through the soil can be directed to the water collection tank through a pre-designed distillation and condensation path inside the acrylic box. To demonstrate the small-scale collection and utilization of low-grade energy by the ISUM array, we conducted a practical application demonstration for about 6 h from 11:15 to 17:04 when the weather was fine. The ISUM array was measured to have a stable output voltage of 1.2 V, which is sufficient for a small LCD spreadsheet to display the time continuously while the generated pure water is collected in the collection tank.

### 3.4. Soil Remediation Performance of ISUM

**Figure 6:**
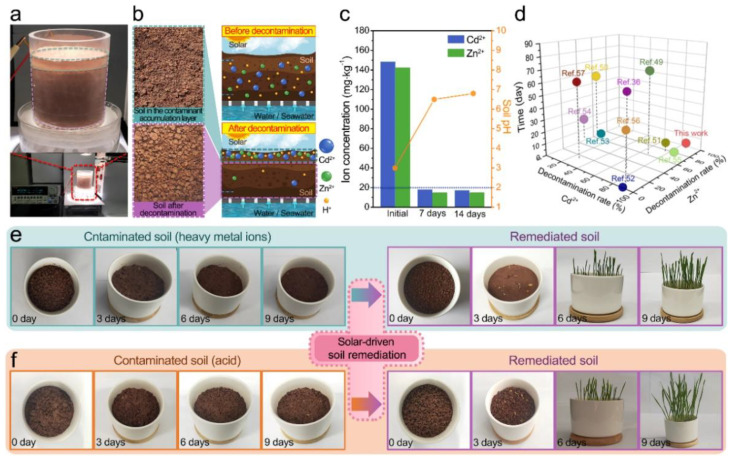
(**a**) Diagram of remediation of contaminated rocky land; (**b**) Photos of the contaminated soil layer enriched with heavy metal ions (top left) and the remediated soil layer (bottom left), and (right) a schematic diagram of the soil remediation process; (**c**) Heavy metal ion concentration in the soil and soil pH before and after remediation (7 and 14 days); (**d**) Plot of Zn^2+^ and Cd^2+^ removal efficiency, removal time of solar-driven ISUM compared to other commonly used techniques; (**e**,**f**) Growth of wheat on different dates (day 0, 3, 6, and 9) in heavy metal and inorganic acid contaminated rocky land compared to remediated rocky land.

Intriguingly, the solar-driven ISUM enables remediation of contaminated soil in addition to generating water vapor and electricity. We pre-prepared two contaminated soil samples for testing, one containing 140~150 mg·kg^−1^ heavy metals Zn^2+^, Cd^2+^, and the other with pH = 3. The ISUM for contaminated soil remediation using solar-driven interfacial evaporation is shown in Figure 6a, and its construction is not different from the schematic in Figure 2b. The schematic in Figure 6b visualizes the solar-driven bottom-up movement of water molecules in the soil, which eventually enriches heavy metal ions (Zn^2+^, Cd^2+^) or acids (H^+^) on the upper surface of the soil. The heavy metal ion concentration plot in Figure 6c and the EDS elemental mapping analysis in Figure S15 compare the heavy metal ion content and pH in the soil before and after remediation. Changes in heavy metal ion concentrations (90% removal) and pH in the soil after 7 and 14 days of solar-driven interfacial water evaporation remediation indicate that remediation of the heavy metal/acid-contaminated rocky land samples in the simulated experiment was nearly complete in only 7 days. The remediated soil with very small amounts of residual heavy metal ions meets the standards for commercial and industrial use (concentration values shown as dashed lines in Figure 6c) and is not harmful to the environment. We compared the removal efficiency (removal rate and time) of ISUM for Zn^2+^ and Cd^2+^ with other typical soil remediation techniques, including phytoremediation (PR), biochar remediation (BR), soil washing remediation (SWR), and enhanced electrokinetic remediation (EER), as shown in Figure 6d. Although PR is environmentally friendly, it often takes up to 40–60 days [36,49]. BR has a maximum removal rate of 70% and is also very time-consuming [50,51,52]. The treatment time of SWR takes only about 120 min to remediate and achieves a removal rate of 80%, but there is some loss of soil nutrients after remediation [53,54,55]. EER has low economic efficiency due to its high cost and energy consumption [56,57]. Compared to other techniques, solar-driven interfacial water evaporation remediation was significantly more effective in terms of remediation time and heavy metal ion removal rate. To more visually compare the effectiveness of the solar-driven interfacial water evaporation soil remediation technique, we selected rocky land with heavy metal ions and acid contamination, and the remediated rocky land as the soil for wheat cultivation. Figure 6e,f show the growth of wheat at room temperature (20 °C) in heavy metal and acid contaminated rocky land and remediated rocky land, respectively. Wheat seeds could not germinate from the beginning to the end in the contaminated soil samples. On the one hand, this is due to the accumulation of heavy metals in the soil organic layer, which affects the biological activity of the soil and slows down the rate of decomposition of soil organic matter, thus inhibiting normal seed development. On the other hand, overly acidic soils can harm the growth of plants. The most important effect of pH in the soil is on ionic solubility, which in turn affects the growth of microorganisms and plants. A pH range of 6.0 to 6.8 is ideal for most crops as it coincides with the optimal solubility of the most important phytonutrients. As a control, wheat seeds planted in the remediated rocky land began to germinate normally on the third day and wheat seedlings grew to 7–8 cm tall by the ninth day. In short, the new concept of soil remediation using solar-driven interfacial water evaporation can achieve the purpose of purifying contaminated soil in a short period of time and meet the basic requirements of soil for industrial and agricultural use.

## 4. Conclusions

The ISUM constructed uses solar-driven interfacial water evaporation combined with soil to simultaneously generate water vapor (pure water), collect low-grade energy, and remediate contaminated soil. By providing an adequate supply of capillary water to the soil, ISUM produced 1.2 kg·m^−2^·h^−1^ of water under 1-sun irradiation and maintained a stable output voltage of 0.3 V for at least seven days. Using the solar-driven interfacial water evaporation technique, 90% of heavy metal ions were removed from the soil after only seven days of continuous exposure to sun irradiation, significantly outperforming other common soil remediation techniques. Finally, plant tests showed negligible soil degradation after treatment. This work not only develops a natural alternative photothermal material, but also provides a new equipment platform for the development of sustainable and environmentally friendly water treatment systems, low-grade energy harvesting, and contaminated soil remediation.

## Data Availability

The data presented in this study are available on request from the corresponding author.

## Supplementary Materials

The following supporting information can be downloaded at: https://www.mdpi.com/article/10.3390/nano12111800/s1, Figure S1: Soil treatment process and photon microscope photographs of six soils; Figure S2: Thermogravimetric (TG) analysis of five soils except shifting sand at temperatures ranging from room temperature to 800 °C; Figure S3: Evaporation rate of gravity water, capillary water and hygroscopic/film water; Figure S4: The surface temperature of different soils under 1 kW·m^−2^ sun irradiation for 1 h; Figure S5: The solar thermal conversion efficiency of six soils; Figure S6: Water transport rates of different particle sizes and thicknesses; Figure S7: Concentration measurement of Cu^2+^ before and after desalination; Figure S8: The calculated equivalent enthalpy and water evaporation rate of pure water and water in rocky land; Figure S9: The mass change of the water with water transport in rocky land and pure water under 1 kW·m^−2^ sun irradiation for 1 h; Figure S10: Stability measurement of evaporation rate of water in rocky land under 1 sun irradiation for continuous 10 h; Figure S11: Water evaporation rate based on different water content and the mass change of soil (30%) outdoor for 6 h; Figure S12: Induced voltage of ISUM under different evaporation rate; Figure S13: Zeta potentials of six soils; Figure S14: Induced voltage and current of a single ISUM consisting of rocky land; Figure S15: Contents of Zn^2+^ and Cd^2+^ in contaminated rocky land and soil remediated for 7 days and 14 days; Table S1: Energy dispersive spectrum (EDS) analysis of initial contents of Zn^2+^ and Cd^2+^ in rocky land contaminated by heavy metals.
