# Supplementary material for: Solar-Driven Soil Remediation along with the Generation of Water Vapor and Electricity
Source: Nanomaterials (Basel). 2022 May 25;12(11):1800. doi: 10.3390/nano12111800 (PMC9182396; doi:10.3390/nano12111800)
Supplement: Supplementary file 1 [file nanomaterials-12-01800-s001.zip › nanomaterials-1686629-supplementary.pdf]

Supporting Information

Solar-driven soil remediation along with the generation of water vapor and electricity

Xiaoting Liu [1,2,3], Zhe Wang [1,2], Hanxue Liang [4], Yuanyuan Li [1,2,3], Tianfu Liu [1,2,*], Qiang Guo [1,2,3], Liru Wang [1,2,3], Ya'nan Yang [1,2,3] and Nan Chen [1,2,3,*]

1 Key Laboratory of Cluster Science, Ministry of Education of China, School of Chemistry and Chemical Engi-neering, Beijing Institute of Technology, Beijing 100081, China; xiaoting_liu2@126.com (X.L.); wzhe1214@gmail.com (Z.W.); yyli3478430@163.com (Y.L.); 3120185630@bit.edu.cn (Q.G.);
example_wlr@163.com (L.W.); yangyanan310@163.com (Y.Y.)
2 Key Laboratory of Photoelectronic/Electrophotonic Conversion Materials, School of Chemistry and Chemical Engineering, Beijing Institute of Technology, Beijing 100081, China
3 Yangtze Delta Region Academy of Beijing Institute of Technology, Jiaxing 314019, China
4 Tianjin Key Laboratory of Biosensing and Molecular Recognition, Research Centre for Analytical Sciences, College of Chemistry, Nankai University, 94 Weijin Road, Tianjin 300071, China; lhxsdb@163.com
* Correspondence: liutf@bit.edu.cn (T.L.); gabechain@bit.edu.cn (N.C.)

Supplementary Note 1: EDS of supernatant of partially dissolved rocky land.

To test the X-ray energy spectrum of ions in rocky land, the solution needs to be made into a powder. The steps are as follows: Weigh 25 g of rocky land, stir and dissolve it with 100 mL of deionized water, then let it sit after ultrasonic for 6 h. The supernatant is placed in the centrifuge tube and centrifuged twice at a speed of 5000 r/min for 5 min each time. After centrifugation, pour the supernatant into a round-bottom flask and connect the flask to a rotary evaporator. Set the rotary steaming temperature to 45°C and steam the water dry. Finally, the round bottom flask was dried in a vacuum oven at 60°C for 5 h to obtain the powder products.

Supplementary Note 2: Preparation of power-generating device.

According to the device shown in Figure 2a, the specific preparation process is as follows: First, fix several absorbent cotton bars with polystyrene foam and place them on a beaker filled with water. Place a filter paper on the foam and an acrylic square frame on top of the filter paper. Then, a layer of soil 1cm thick is placed inside the square frame, and a cut-titanium mesh is placed on the soil, which is connected to a silver wire that extends to the test instrument through a hole in the frame wall. A certain thickness of soil was laid on the titanium mesh electrode, depending on the requirements of subsequent experiments and then place a titanium mesh electrode. Finally, a final layer of soil was laid on the titanium mesh electrode to complete the preparation of ISUM.

Supplementary Note 3: The mechanism of generating electrical signals.

Solar-driven interfacial evaporation not only collects pure water, but also generates an electrical signal. In ISUM, like transpiration in plants, as water evaporates, the water in the dark colored soil is driven by the solar thermal effect from the bottom to the top of the soil, resulting in a stable and persistent electrical signal output. Two inert metal mesh electrodes were placed at a distance in the soil at ISUM to measure the induced voltage and current (the electrodes were made of titanium mesh and connected to a digital source meter Keithley 2400 using silver wire) (Figure 2b). Four different particle sizes of rocky land were selected to investigate the differences in the induced electrical signal (Fig. 3b). The test results show that the voltage value increases first and then decreases for the rocky land with increasing particle size. The reason is that soils with smaller particle size and larger specific surface area have higher capillary action, resulting in a higher specific gravity of bound/capillary water within the soil and less water mobility. And hydronium ions, produced by the interaction between water molecules and the negative charge on the surface of the soil, cannot flow freely in a directional direction, resulting in a smaller potential difference between the electrodes and therefore a smaller inductive electrical signal. On the contrary, the larger the particle size is, the larger the water channel formed between the particles is, and the hydronium protons attached to the surface of soil particles can freely flow upward. But the weak capillary action leads to the insufficient interaction between water molecules and the soil surface, resulting in fewer hydronium protons, so the potential difference between electrodes is small. Figure 2c and 3b indicate that evaporation and electricity generation performance of rocky land with 630 μm is better. Therefore, the soil samples used in the following experiments on electricity generation are rocky land with 630 μm.

Supplementary Note 4: Zeta potential for other soil.

The Zeta potential test requires a solution with a concentration of PPM, so in this case we prepare solutions of 5 soils (0.1% mass) in addition to shifting sand. Since shifting sand is insoluble in water, we use a high-speed ball mill to treat it at 60 RPM for 48 h. Grind the right amount of shifting sand and mix it into a solution (0.1% mass). Finally, the Zeta potentials of these soils were measured.

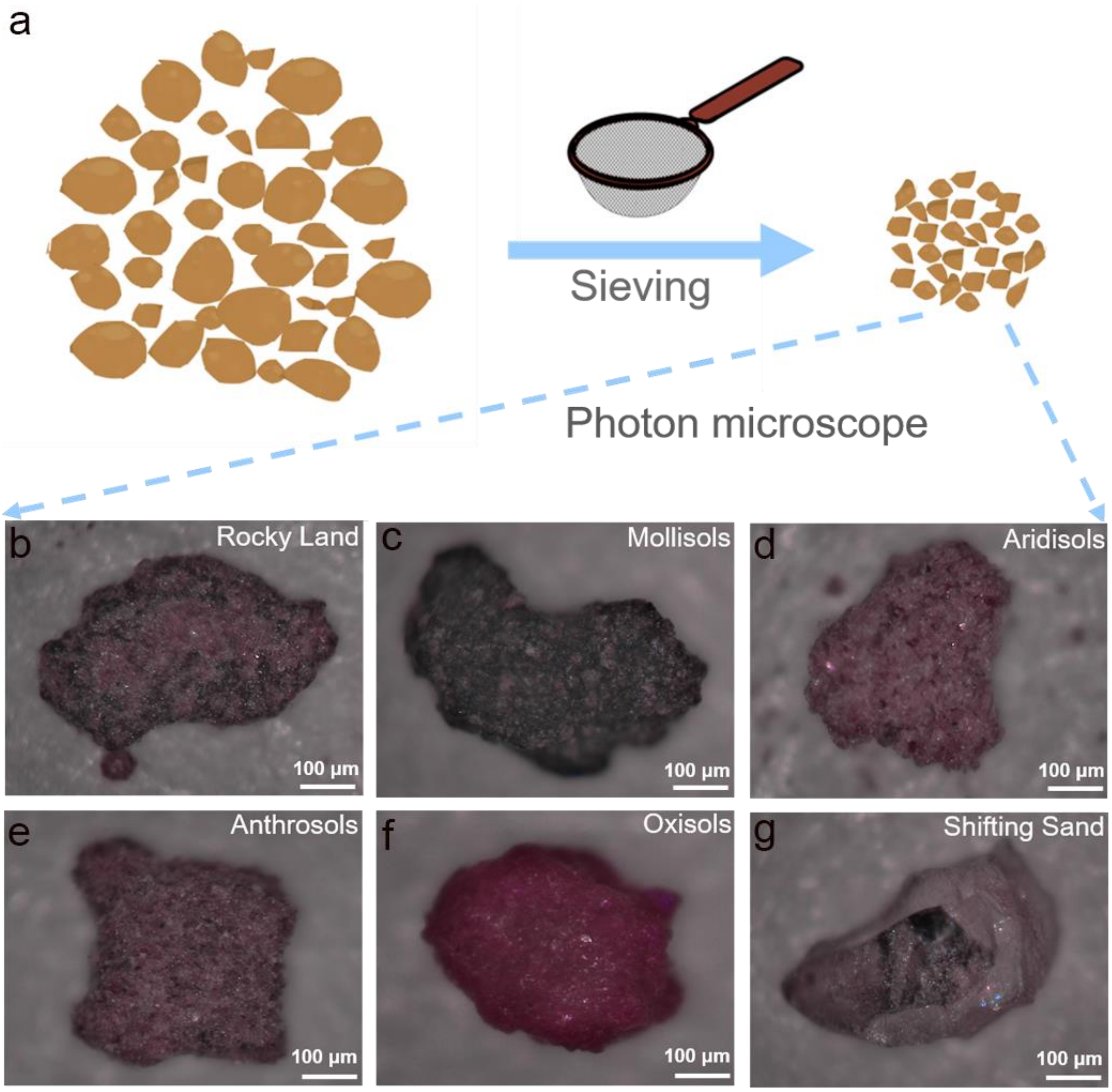

Figure S1. (a) Soil treatment process and photon microscope photographs of (b) rocky land, (c) mollisols, (d) aridisols, (e) anthrosols, (f) oxisols, and (g) shifting sand.

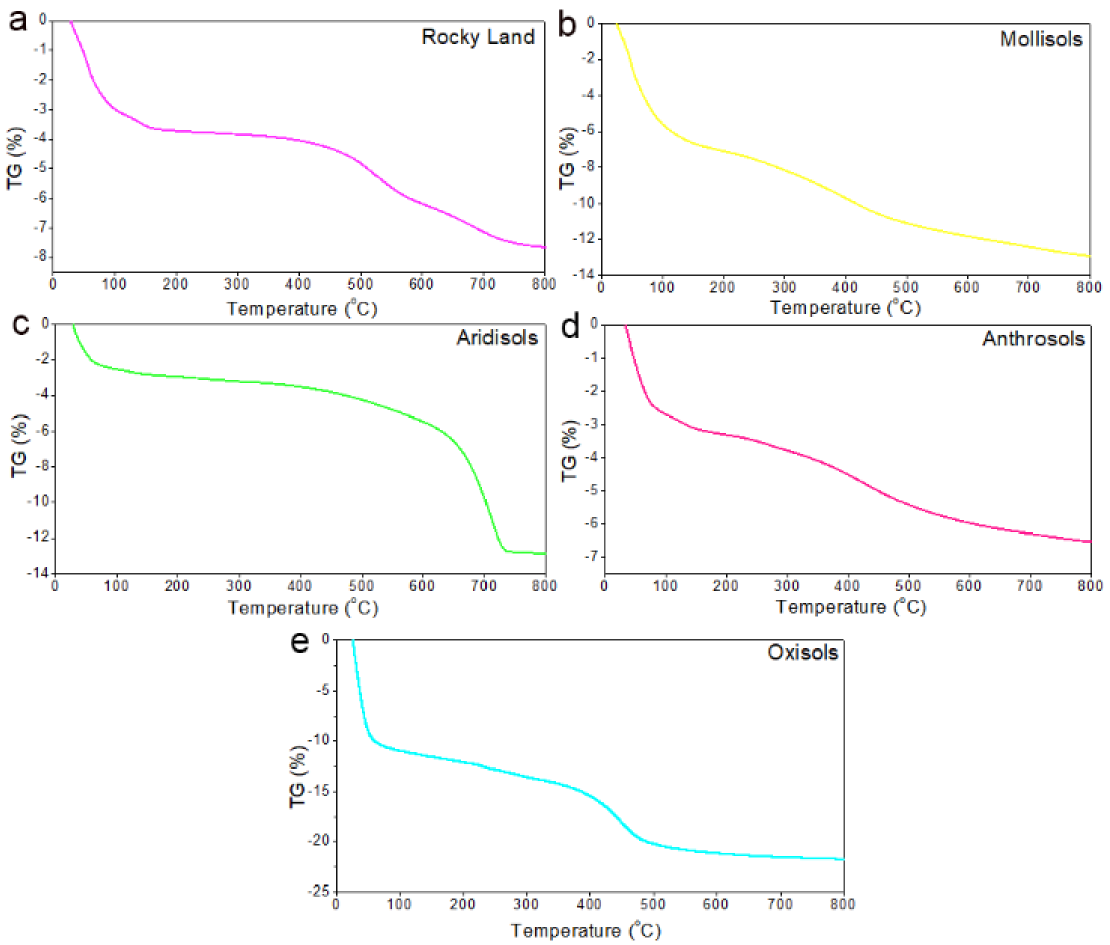

Figure S2. Thermogravimetric (TG) analysis of five soils except shifting sand at temperatures ranging from room temperature to 800°C. The shifting sand is so stable that there is no mass change in the tests. TG of (a) rocky land, (b) mollisols, (c) aridisols, (d) anthrosols, and (e) oxisols.

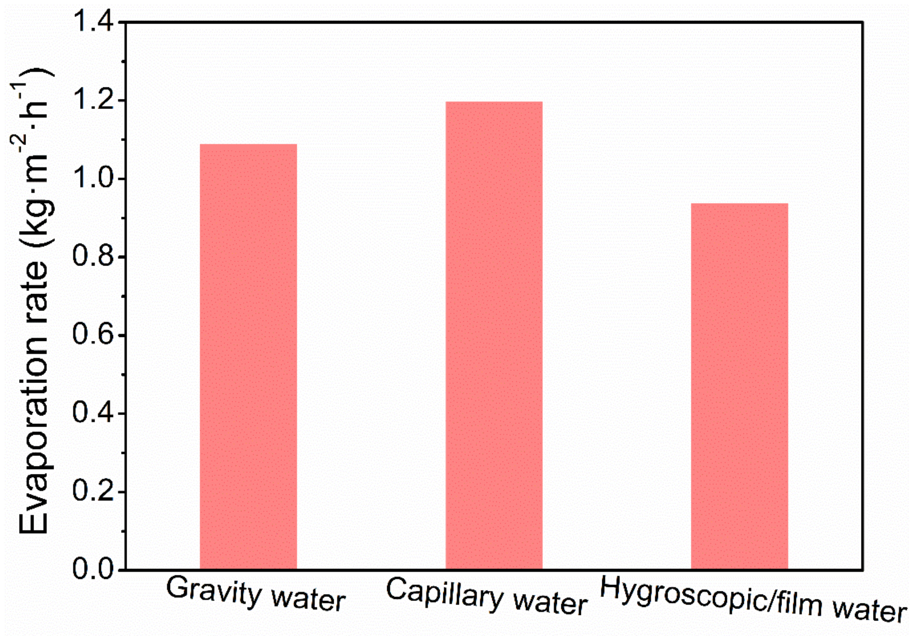

Figure S3. Evaporation rate of gravity water, capillary water and hygroscopic/film water.

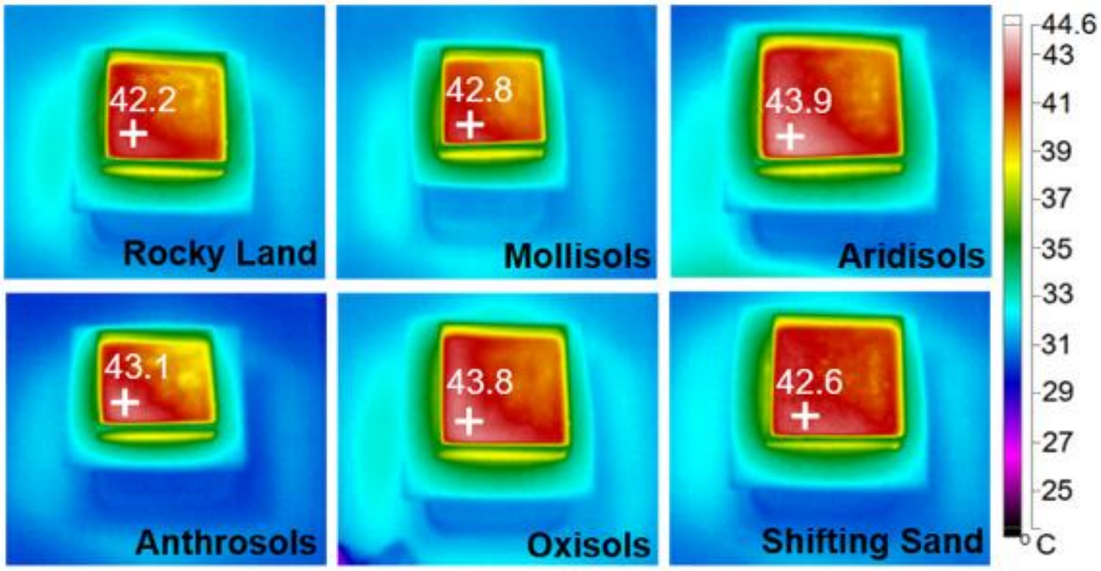

Figure S4. The surface temperature of different soils under 1 kW·m^{-2} sun irradiation for 1 h.

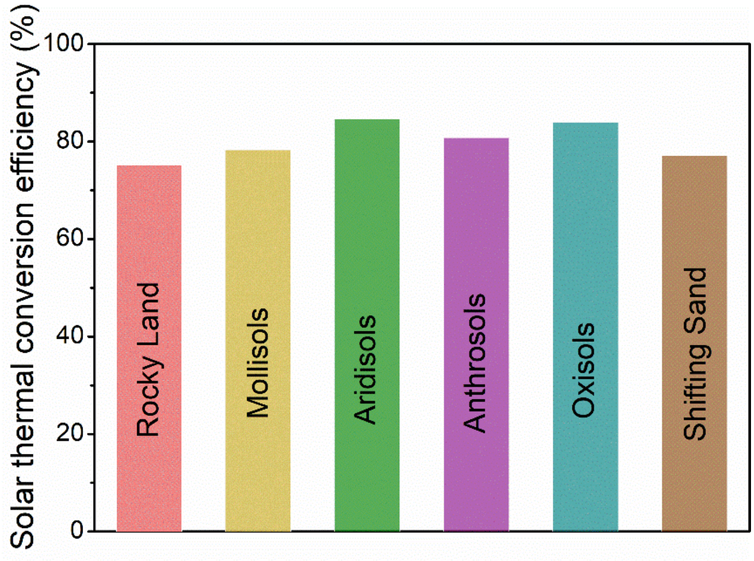

Figure S5. The solar thermal conversion efficiency of six soils.

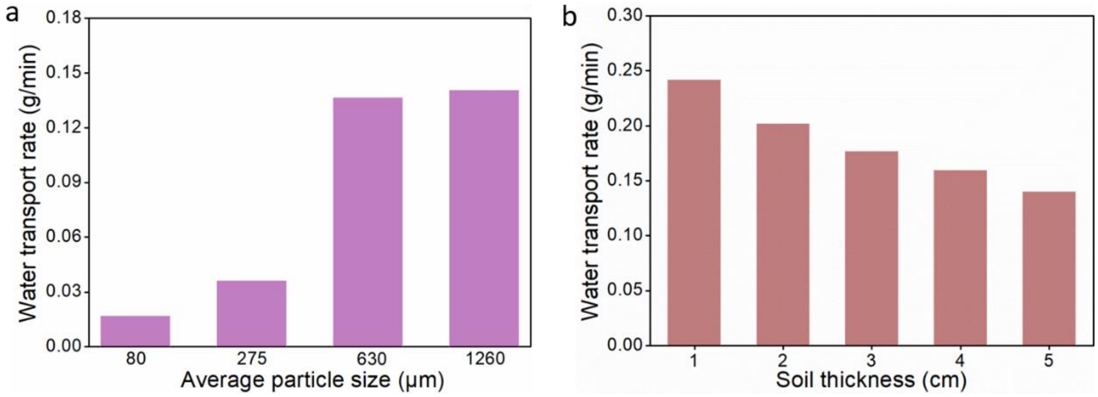

Figure S6. Water transport rates of different particle sizes (a) and thicknesses (b). Water conductivity is the amount of water the soil absorbs per minute [1].

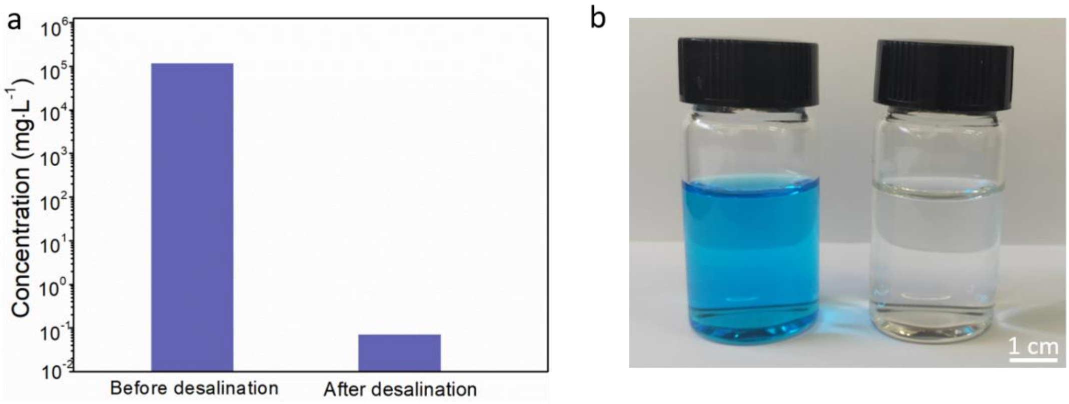

Figure S7.(a) Concentration measurement of Cu^{2+} before and after desalination. (b) Photograph of water collected after desalination and salt solution before evaporation.

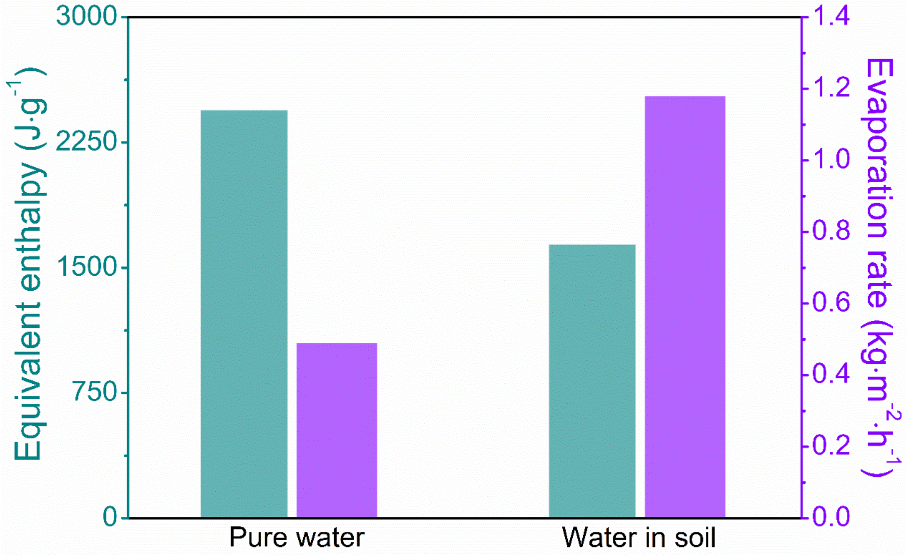

Figure S8. The calculated equivalent enthalpy and water evaporation rate of pure water and water in rocky land.

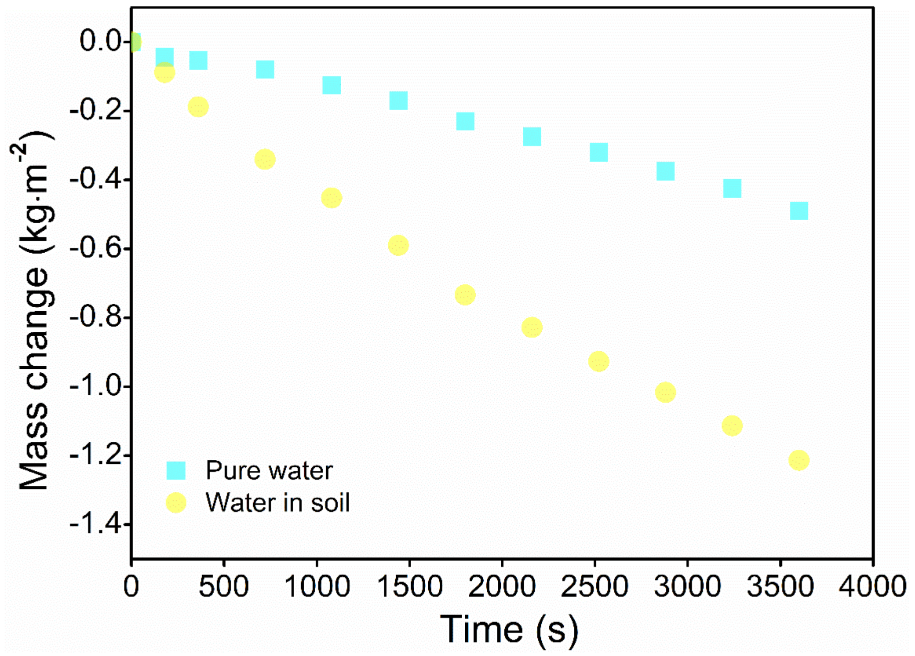

Figure S9. The mass change of the water with water transport in rocky land and pure water under 1 $kW{\cdot}m^{-2}$ sun irradiation for 1 h.

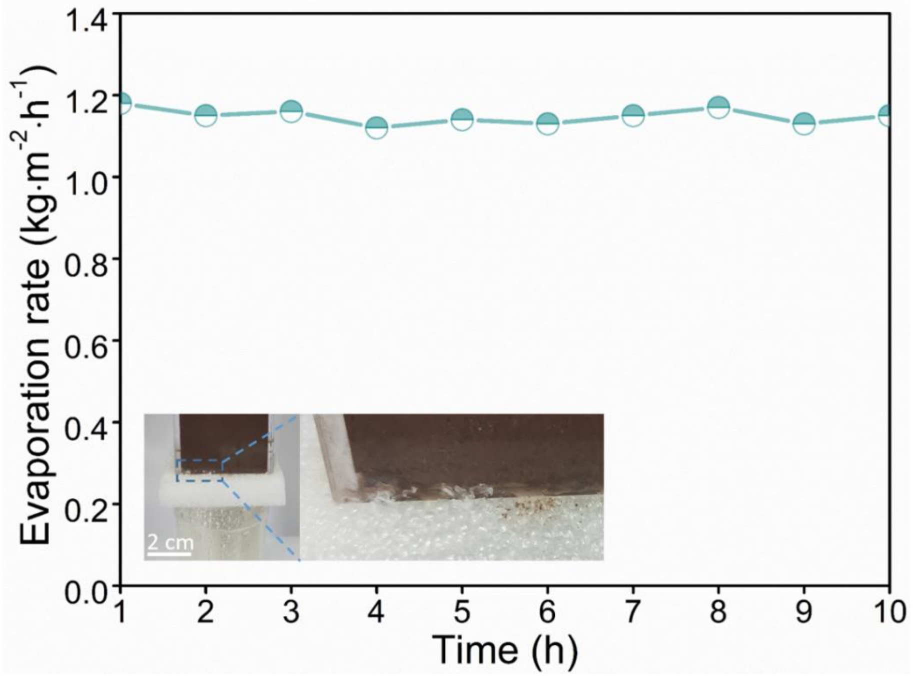

Figure S10. Stability measurement of evaporation rate of water in rocky land under 1 sun irradiation for continuous 10 h (the inset: salt crystals after water evaporation for 10 h).

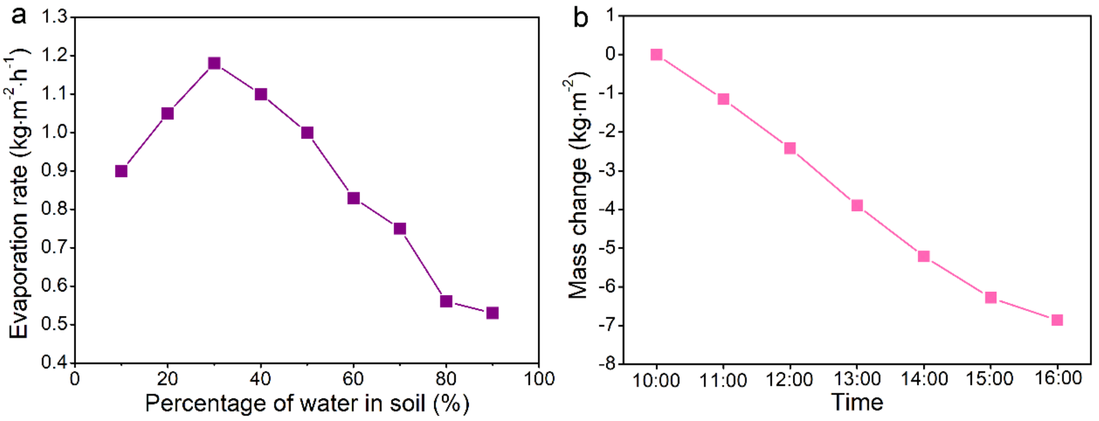

Figure S11. (a) Water evaporation rate based on different water content. (b) The mass change of soil (30%) outdoor for 6 h.

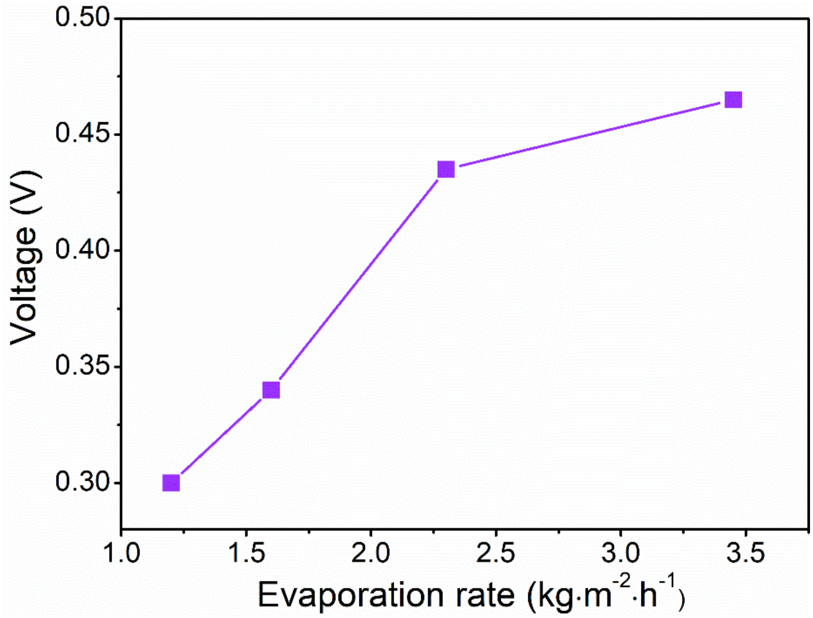

Figure S12. Induced voltage of ISUM under different evaporation rate.

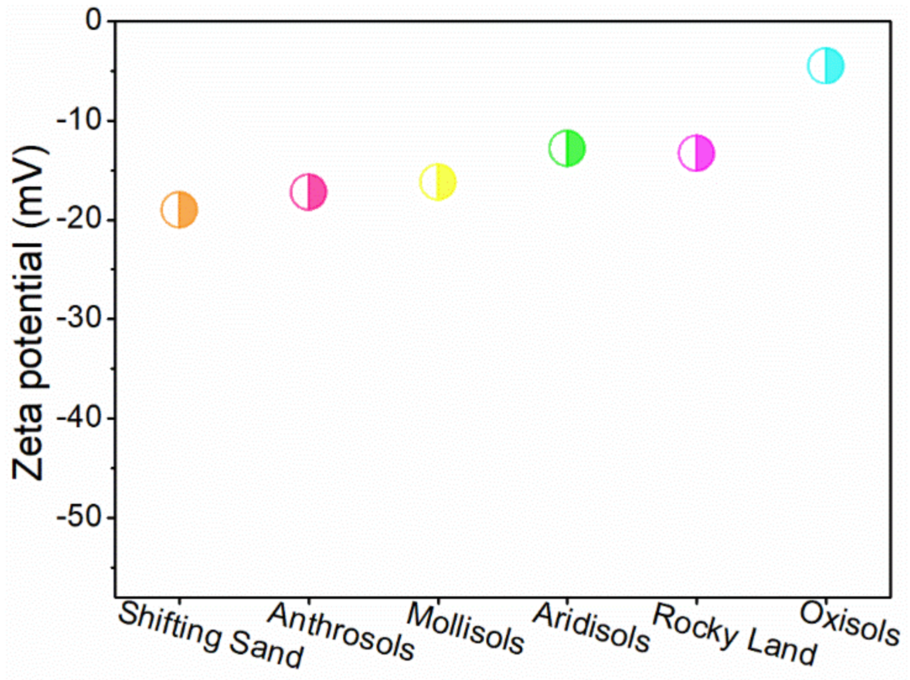

Figure S13. Zeta potentials of the shifting sand, anthrosols, mollisols, aridisols and oxisols are -19, -17.2, -16.2, -13.3 and -4.5 mV, respectively. According to the study of Zeta potential of oxides Al_2O_3 (2.5 V), Fe_3O_4 (-1.0 V), SiO_2 (-1.7 V) [2], the Zeta potential of the other five soils is likewise negative.

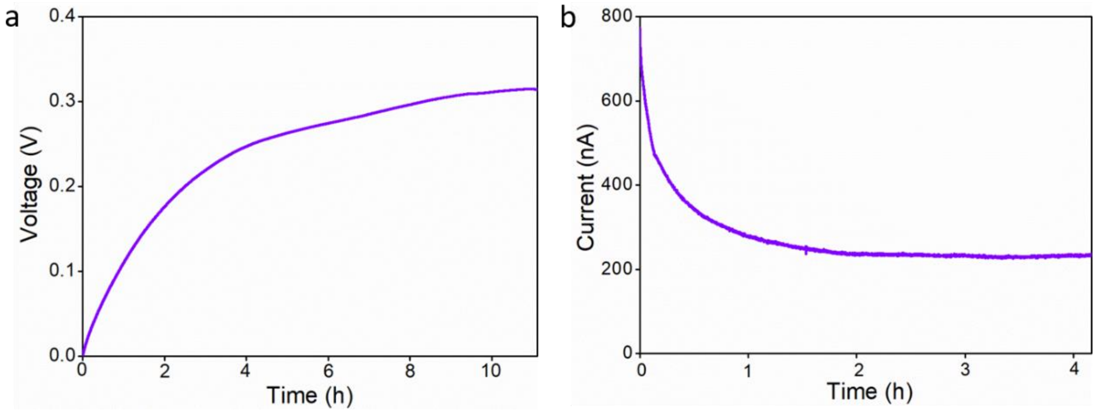

Figure S14. (a) Induced voltage of a single ISUM consisting of rocky land. (b) Induced current of a single ISUM consisting of rocky land.

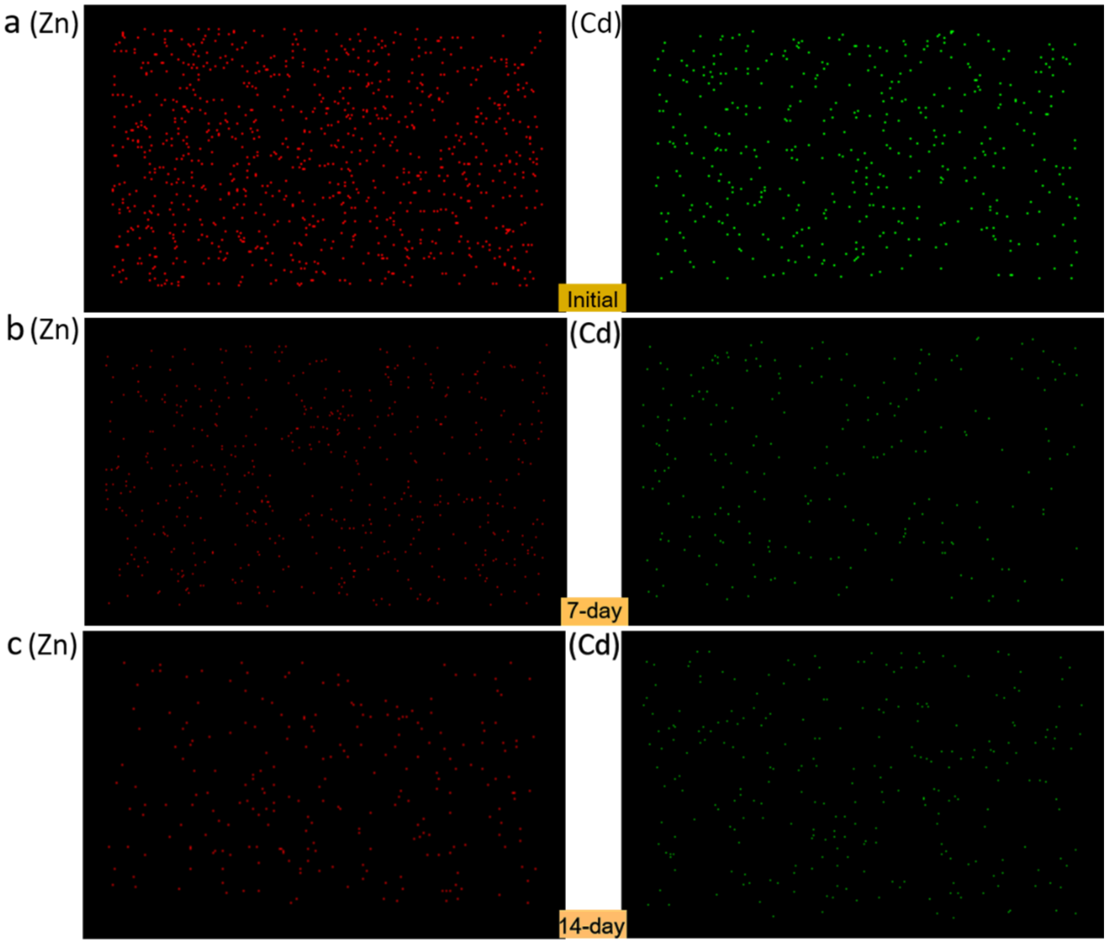

Figure S15. Contents of Zn^{2+} and Cd^{2+} in (a) contaminated rocky land and soil remediated for (b) 7 days and (c) 14 days. It can be seen from elemental mappings that Zn and Cd contents are significantly reduced after purification for 7 and 14 days respectively.

Table S1. Energy dispersive spectrum (EDS) analysis of initial contents of Zn^{2+} and Cd^{2+} in rocky land contaminated by heavy metals.

Element	Weight %	Atomic %
**C**	57.71	69.08
**O**	24.67	22.17
**Al**	4.13	2.20
**Si**	12.49	6.39
**Zn**	0.32	0.07
**Cd**	0.68	0.09

References

[1] Guo, Y.H.; Lu, H.Y.; Zhao, F.; Zhou, X.Y.; Shi, W.; Yu, G.H. Biomass-Derived Hybrid Hydrogel Evaporators for Cost-Effective Solar Water Purification. *Adv. Mater.* **2020**, *32*, 1907061.

[2] Shao, C.X.; Ji, B.X.; Xu, T.; Gao, J.; Gao, X.; Xiao, Y.K.; Zhao, Y.; Chen, N.; Jiang, L.; Qu, L.T. Large-Scale Production of Flexible, High-Voltage Hydroelectric Films Based on Solid Oxides. *ACS Appl. Mater. Interfaces* **2019**, *11*, 30927–30935.
